# Observations on Aneurism and Its Treatment by Compression

**Published:** 1847-11

**Authors:** 


### BUFFALO MEDICAL JOURNAL

AND

### MONTHLY REVIEW.

VOL. 3. NOVEMBER, 1847. NO. 6

### ORIGINAL COMMUNICATIONS.

ART. I.—*Observations on Aneurism, and its Treatment by Compression.* By O'Bryen Bellingham, M. D., Edin. Fellow of and Professor in the School of the Royal College of Surgeons in Ireland; Licentiate of the Royal College of Surgeons of Edinburgh; and one of the Surgeons to St. Vincent's Hospital. Printed at the Medical Press Office, 15 Molesworth-st., Dublin. 12 mo. From the Author.

During the last year or two, the foreign Medical Journals have been much occupied with the subject of curing external aneurisms by moderate pressure applied remote from, and upon the cardiac side of the aneurismal tumor. A considerable number of cases have already been reported, and farther experiments are still in progress. Meanwhile, surgeons have not all been agreed upon the merits of the new plan, but have begun to record themselves as friends or antagonists; some defending it with facts and philosophy, and others assailing it as an old system, which, having been thoroughly tried, was long since abandoned; or which, if such were not the case, is far inferior in point of certainty, ease and safety, to the operation of Hunter. Those, however, who find any thing to say against the system, are, as yet, comparatively few. It, thus far, seems to be generally conceded, that whether the system is old or new, a question in itself of very little moment, it does appear to effect radical cures in a mode at once safe and simple, though occasionally, somewhat tedious; and we have just reason to believe that a portion, at least, of the opposition which exists, is the result of some sectional jealousies, or of an unwillingness to see surgery forcably deprived of one of its most brilliant and imposing operations, viz.; the application of the ligature to the aneurismal artery.

After a careful retrospect of the whole matter, we must confess that we have fallen in with the new plan—new at least to us, in so far as the mode and degree of pressure is concerned; and we hail the operation as another triumph of conservative surgery.

It is with pleasure, therefore, that we now call attention to this first publication, *in extenso*, which we have seen upon this subject, and from a gentleman who was himself one of the first to make a successful experiment, and who has since either made, or witnessed several more. Mr. Bellingham resides at Dublin, to the surgeons of which city the operation was for some months wholly confined, and where its merits and demerits have been more critically and severely examined than any where else. The author has, also, all along, been very much devoted to the subject, having at an early date become involved in a controversy as to the mode in which pressure, applied remote, and upon the cardiac side, effects the obliteration of the aneurismal sac.

The first five chapters of the book before us are devoted, chiefly to the history of the cure of aneurism by compression, from the earliest day down to the present time; and contains much information, and many cases, which have not before been brought together; an analysis of the whole of which shows all the following modes to have been at one time or another practised:—

"1. Where the pressure was applied directly upon the aneurismal sac.

2. Where the pressure was applied to the aneurismal sac, and the entire limb was at the same time compressed.

3. Where the compression was applied to the artery between the aneurismal sac and the heart.

4. Where the pressure was applied to both the aneurismal sac and the artery upon the cardiac side.

5. Where the pressure was applied to the distal or capillary side of the aneurismal sac.

6. Where the aneurismal sac was laid open, and the pressure was applied upon the ruptured or wounded vessel.

7. Where the artery above the aneurism was laid bare, and the pressure was applied directly upon it."

It is the plan adopted in the third section which some say is now *revived*, but which the author aims to show is not the new practice. Galen, who lived in the second century, is said to have cured an aneurism at the bend of the arm by means of a sponge and bandages, and Rhazes, of the ninth century, is stated to have recommended similar measures. Jean de Vigo, a physician of Genoa, who lived in the begining of the 16th century, is known to have treated aneurisms by compression, styptics, &c., and a distinct case is recorded by Tulpius, of Amsterdam, who wrote in 1641.

From this date up to the time of Hunter, the history of the various modes of treating aneurism is easily traced. Compression had been tried in many forms, and had signally failed, and so, also, had the operation of opening the sac and tying above and below, so that Pott, who wrote in 1777, condemns all operations made to cure an aneurism, for he declares he had never seen any other operation than that of amputation which had preserved the life of the patient.

In 1785, Hunter performed his first operation of tying the popliteal artery remote from the sac, and on the cardiac side. The great superiority of this to all previous modes was soon generally established; yet there were a few surgeons who never entirely abandoned the old methods—thus, even as late as 1818, Boyer says, "in certain cases of popliteal aneurism the old operation is preferable to the Hunterian." By the "old operation," Boyer meant the operation of exposing the sac itself, and tying, above and below. Pressure, also, continued to be employed in a few rare cases, but it was thereafter no longer made upon the sac, but only between the aneurism and the heart. The first case of cure reported from this plan of treatment, occurred in Paris, in 1810, under the care of M. Eschard Several others followed, and, as some were cured, and others were compelled to abandon the pressure and resort to the ligature, Dupuytren, who had witnessed several of the cases, concluded in 1829, "that there are individuals who cannot bear compression, whilst there are others who support it without inconvenience," &c.

It must now be understood, that up to this time, and later, compression was applied to the artery on the cardiac side of the sac, with a view of *obliterating the artery* at the point selected, and thus effecting a cure of the aneurism below; and the difficulty consisted chiefly in the inability of the patients to endure such an amount of pressure as was necessary to accomplish this result. Whereas, the principle involved in the plan now proposed, requires that the artery leading to the sac *shall not be entirely closed, but only sufficiently to materially diminish the quantity of blood flowing through it.*

We can scarcely doubt that compression as formerly used, while it occasionally wrought cures. by completely closing and finally obliterating the artery at the point of pressure, did, also, in some instances, where the pressure was less complete, effect a cure by partially arresting the current. It is not, therefore, so much a question in the history of the treatment of aneurisms, *when the first cure was effected* by partial pressure as *when first the important principle was discovered* by which the cure was effected. We may then pass over that portion of the work before us in which the claims of surgeons to priority in this mode of treatment are reviewed, and come directly to the period when Mr. Bellingham himself first explained its *modus operandi*, and declared his conviction of its superiority to all other previous plans.

This was at a meeting of the surgical society of Ireland, held in Dublin, on the 22d of April, 1843, a report of the proceedings of which is contained in the Dublin Medical Press, published in the May following. At this meeting, three cases of cure of aneurism by pressure on the cardiac side of the sac were reported, one by Mr. Hutton, one by Mr. Cusack, and the third by Mr. Bellingham; and on this occasion, Mr. Bellingham made the following remarks:

"Surgical writers appear to have been under the impression, that in order to cure an aneurism by compressing the artery above the tumour, it was essential tointerrupt completely the current of blood through the vessel—in fact, to apply such pressure as would act like a ligature, cause inflammation of the coats of the artery at the part, and obliterate the circulation in the vessel at the point to which compression had been applied.

When it was considered obsolutely necessary for the success of compression, that such an amount of pressure should be applied as was almost certain to produce sloughing of the part and very certain to occasion intense pain and suffering; and when, in addition, this was to be prolonged through five successive nights and days, [as in the case reported by Mr. Guthrie which I had quoted,] we can readily understand why patients refused to submit to it, and we can easily account for the disrepute into which the practice fell, and for the unwillingness of surgeons to adopt this treatment, in preference to the simple operation of placing a ligature upon the femoral artery. It would, however, appear that it is not at all essential the circulation through the vessel leading to the aneurism should be completely checked, but rather the contrary: it may, perhaps, be advantageous at first, for a short period, by which the collateral circulation will be more certainly established; but the result of this case, if it does no more, establishes the fact, that *a partial current through an aneurismal sac will lead to the deposition of fibrine in its interior, and cause it within a few hours to be filled and obstructed, so as no longer to permit of the passage of blood through it.* Pressure, so as altogether to obstruct the circulation in an artery, must necessarily be slower in curing an aneurism, as it must, in some measure, act by causing obliteration of the vessel at the part to which the pressure has been applied; whereas *a partial current through the sac enables the fibrine to be readily entangled in the parietes of the sac in the first instances, and this goes on increasing until it becomes filled;* the collateral branches having been previously enlarged, the circulation is readily carried on through them."

The instruments which have already been employed for the purpose of diminishing the current of blood through the artery, have been almost as various as the operators, and we have scarcely a doubt on looking over the several varieties of "clamps," and "screws," and "tournequets," and "trusses," and "compresses,' and "weights," to which the surgeons have so far resorted, that it still remains for some ingenious *yankee* to "invent" the instrument which is to perfect the discovery! These instruments all resemble each other in being designed to make a moderate and endurable amount of pressure upon the artery. They are also so constructed as that they may be relaxed whenever the comfort of the patient demands it; and moveable, so that the pressure may be alternately made at different points in the course of the artery, In some, this last indication is answered by a double headed compressor, one of whose heads is generally loosened when the other is tightened. Mr. Carte has invented an apparatus by which he is enabled to apply a *weight* to the walls of the vessel; the weight resting in a kind of "hopper." To make the requisite pressure upon the femoral artery of an adult from seven to nine pounds are sufficient.

We shall enable the reader better to understand the new practice by giving a running epitome of the twenty-seven cases reported as having been submitted to the new treatment.

Case 1. A laborer, aged 30. Popliteal aneurism about the size of a hens' egg. In twenty-eight days after the compression was commenced, the pulsation in the aneurism ceased. The compression was continued three days longer; and a month later the patient was discharged, the tumor being now of the size of a nutmeg, and hard; pulsation of femoral artery continued natural. *Mr. Hutton, of the Richmond Surgical Hospital, operator.*

Case 2.—A tanner, aged 55. Ppopliteal aneurism, size of hen's egg. Pressure continued upon the artery in the groin four weeks and three days; pulsation having ceased at the end of four weeks. Femoral artery at the last dates continued to pulsate freely. Tumor reduced to size of a walnut, and very hard. *Mr. Cusack. of St. Stevens' Hospital, operator.*

Case 3.—Servant, aged 32. Popliteal aneurism; about three inches in diameter. Pressure applied upon the artery as it passes over the ramus of the pubis, April 3d, discontinued on the 4th and 5th, reapplied on the 6th, and again on the 11th. The absolute time occupied in the compression was *two days*. Pulsation in tumour ceased on the 7th of April. One month after, the tumor was very small; the pulsation of femoral artery continued, and patient was pronounced cured. *Mr. Bellingham operator.*

Case 4.—A tinplate worker, aged 30. Femoral aneurism. Compression made at lower part of upper third of the femoral artery Continued fifty-six days, pulsation in the tumor having ceased six days previously. The day before the pulsation ceased, he felt great pain in the limb; scarcely any tumor left. *Mr. Liston, of London, operator.*

Case 5.—A carpenter, aged 29. Popliteal aneurism. Compressed ninety-three days; applied at first upon the ramus of the pubis, and afterwards about four inches below; pulsation in tumor ceased after about three months. Completely cured. *Mr. Harrison, of Jervis-street Hospital, operator.*

Case 6.—Aged 53. Femoral aneurism, sixteen inches in its greatest circumference. Compressed thirty days; and on the fourteenth day after the instrument was removed, the report says the tumor is smaller, and the case promises very favorable, the pulsation has ceased. *Mr. Liston, operator.*

Case 7.—Servant aged 33. Femoral aneurism, two inches in diameter. Compression applied over ramus of pubis, made at intervals, continued 43 days; tumor then size and shape of a small hen's egg, very firm and solid. *Mr. Bellingham, operator.*

Case 8.—A baker, aged 28. Popliteal aneurism, size of hen's egg. Compressed fifty-three days, shortly after which, it is reported, "a small firm nodule marks the situation of the aneurism, and the patient is discharged cured, but his limb is yet stiff, weak, and somewhat emaciated." *Mr. Kirby, of Jervis-street, operator.*

Case 9.—Popliteal aneurism; cured in fifty-seven days, by *Mr. Allan, of Haslar Hospital.*

Case 10.—Popliteal aneurism; cured in twenty-one days, by *Mr. Greatrex, of London.*

Case 11.—Popliteal aneurism; treated with two instruments, one applied upon the femoral artery at the lower part of Scarpa's space, the other higher up the limb. Compression continued seven days; tumor was very large, but now is entirely gone. *Mr. Cusack, operator.*

Case 12.—Popliteal aneurism; two instruments used; one applied about two inches below Poupart's ligament, the other near the tendinous canal in the triceps muscle. Cured in twenty-four days. *Mr. Porter, of Meath Hospital, operator.*

Case 13.—Aged 30. Popliteal aneurism, has disease of heart, sac thin. Duration of compression twenty days, when the patient suddenly died. The pulsation in sac had ceased two days previous. *Mr. Cusack, operator.*

Case 14.—A medical man; popliteal aneurism; two inches in diameter. "Compression was commenced August 2nd, continued for twenty days, when the patient suffered so much from a burning sensation along the course of the tibia, that he refused to continue it, and removed to the country; there was then 'a decided hardness in the seat of the disease, but the pulsation appeared nearly as great as ever.' Four weeks after the discontinuance of the compression, (during a fortnight of which the patient had used much exercise,) an unusual degree of stiffness was felt in the knee, and on examination, the pulsation in the aneurism was found to have ceased, and there was increased hardness in the tumor: no return of pulsation subsequently. Period which intervened between the commencement of the compression and the cure of the aneurism forty-eight days, during the last twenty-eight of which pressure was not applied.

Case 15.—Popliteal aneurism, cured in thirty-three days by weights, applied part of the time about the middle of the thigh, and part of the time in the groin. Treated by *Mr. O'Ferrall, of St. Vincent's Hospital.*

Case 16.—Popliteal aneurism, cured by a tournequet in forty days, by *Mr. Jolley, of the Torbay Dispensary.*

Case 17.—Popliteal aneurism, "Compression commenced with a single instrument; pressure applied to the artery in the groin; a second instrument subsequently used, and the pressure applied to the artery lower down alternately with the other, two days after which the pulsation in the aneurism ceased." *By Mr. Macdonnell, of the Richmond Hospital.*

Case 18.—Popliteal aneurism. Compressed seven days, two instruments applied at same time, one to the groin and the other lower. Cured. *Mr. Dartnell, of the army, operator.*

Case 19.—Femoral aneurism, cured in thirty-six days, by *Mr. Mackern.*

Case 20.—Popliteal aneurism. Compressed by two instruments ninety-one days; during which time the pulsation ceased, and a few months after, the report says: "the tumor is very firm and decreasing in size." *Mr. Stork, operator.*

Case 21.—Popliteal aneurism, cured in twenty-one days by two instruments applied at different points at the same time. *Mr. Stork, operator.*

Case 22.—A medical man. Popliteal aneurism. "Compression commenced March 2nd, followed by numbness about the ham, œdema of the leg and foot, with pain and diminished temperature in the same part. Compression discontinued April 14th, the tumor had become more solid, was diminished in size, but pulsation could still be detected. A compress of sponge was then applied to the tumor, and retained by a bandage.

The patient returned to the country, and commenced taking exercise. In riding a spirited horse on the 22nd of May following, he made a sudden exertion, which was followed by great pain in the knee and tumor, which increased at night. He took a full opiate which procured sleep, and on awaking the pulsation in the aneurism was found to have ceaseed. By the 27th of June the tumor could hardly be felt, but some pain in leg and ankle remained with stiffness in the knee; a disagreeable numbness was also felt along the inner side of the knee, leg, and foot, "probably (the patient thinks) caused by some injury which the saphena nerve incurred during the process of applying the pressure." Compression continued for forty-three days; aneurism ceased to pulsate eighty-one days after the pressure was first used."

Case 23—Popliteal aneurism. Pressure made in the groin by a weight, and in lower part of thigh by a weight. "Compression commenced July 30th at 11 o'clock a. m. by two instruments, which were alternately relaxed, (the pressure being sufficient to check completely the pulsation); at 9 o'clock p. m. very severe pain in the ham set in, the patient unscrewed the instrument, and found that all pulsation had ceased. Compression continued until August 2nd, the pulsation did not return, slighter pressure was then kept up until the 8th, when the instrument was removed. The aneurismal tumor now (September 24th) is about the size of a hen's egg and hard. Duration of compression ten hours; the previous pressure must, however, be taken into account, which had evidently caused deposition of fibrine in the aneurismal sac, because it could not be completely emptied by pressure upon the artery above." *Mr. Sunter, of Newbridge, operator.*

Case 24.—Popliteal aneurism. "Compression commenced soon after the patient's admission; pressure made by a weight in the groin, and by a clamp upon the artery at the junction of the middle with the lower third of the thigh. After the compression had been continued for some time, as the pulsation continued to be strong, it was resolved to give a trial to galvanism combined with compression. By applying pressure upon the artery above and below the aneurism, so as to retain the contents of the sac until acted on by the galvanic current, it was expected that one of the principal causes of the failure of this proceeding would be avoided; the case likewise seemed to be a favorable one, in this respect that the blood contained a very large amount of serum in proportion to the fibrine.

April 21st. A clamp was applied upon the artery above the aneurismal sac, and another below it; two acupuncture needles (insulated except at their points and hafts) were then introduced from opposite sides into the aneurismal sac, and brought into connection with a Smee's battery by Dr. Apjohn, Professor of Chemistry to the Royal College of Surgeons, who kindly afforded his services, and the galvanic current was maintained by him for about fifteen minutes at intervals. It was intended to repeat the application after a short interval, and in the meantime the patient continued the compression. In order to hasten the cure (as he thought) he had kept up very strong pressure upon the artery in the thigh for many hours, when seven days after the employment of the galvano-puncture, he was seized with a shivering, erysipelas (which was prevalent at the time) attacked the part of the thigh upon which the pad of the instrument rested, it spread upwards and downwards, and the patient died on the 4th of May, six days afterwards.

Case 25.—Successful treatment of a case of popliteal aneurism at St. Vincent's Hospital, particulars not yet made public.

Case 26.—Is a case of popliteal aneurism cured by pressure upon the sac itself, and does not belong, therefore, to the class of operations of which we are now speaking.

Case 27.—"Patient, a drover, aged 42, healthy, admitted into the Bristol Infirmary, under Mr. Harrison, September 28th, 1843, laboring under popliteal aneurism on the left side. Tumor had been noticed some months previously; it had attained the size of a large orange; pressure upon the artery in the thigh caused it to diminish. Compression commenced October 3rd, the pressure applied to the artery in the groin. Treatment persevered in for a fortnight, when it was discontinued at the patient's request, and the operation performed. At this period pulsation was distinctly felt in the articular arteries about the knee, when the pulsation was arrested in the femoral artery by compressing the artery in the groin."

Chapter 7th treats of the employment of chemical agents used to coagulate the blood in an aneurismal sac, the employment of heat, galvano-puncture, injections of chemical fluids, &c., &c. Sir Everard Home reports a case in which he employed heated needles to produce a coagulum in 1826. The result was a cessation of pulsation in the tumor; but forty-six days after the last trial, the patient died of gangrene in the diseased limb. The autopsy showed a complete coagulum filling the sac. No other case is reported.

In 1832, Mr. Phillips proposed to obliterate arteries by transfixing them with needles and then passing a galvanic current through them. Seven cases are reported, of which only three proved successful, and we shall dismiss the whole subject with the following conclusions drawn by Mr. Bellingham:—

"On the whole it appears to me that much can never be expected from galvano-puncture as an agent in the cure of aneurism: it is doubtful if the current of a battery of moderate strength, however long continued, can develop a coagulum sufficiently firm to produce even the effects I have mentioned; and the employment of a powerful battery is not without risk if the aneurismal sac is of considerable size, or springs from a large artery. In addition, it is difficult to isolate completely the shaft of the acupunctnre needles; the substance used for this purpose is either detached in the act of introducing them, or it is softened when the galvanic current is completed: hence the tissues through which the needles pass are acted on, and the strength of the current is necessarily wasted. I need hardly delay here to allude to an objection urged against this measure by a writer in the *Dublin Quarterly Journal of Medicine*—viz., that erysipelas may follow the employment of the acupuncture needles, because every one, I presume, is aware that erysipelas may follow any form of puncture, wound, or incision, in any part of the body."

Mr. Wardrop suggests injecting the sac with acetic acid, a small quantity of which will coagulate a considerable mass of blood, but no one has yet put his suggestion into practice. All the above modes are spoken of as possible auxiliaries to compression, whose value may not yet be fully determined.

We shall conclude this brief review of this most interesting little volume, with the writers own summary of some of the most material points relating to this new mode of treatment:—

"1. The arteries to which compression is applicable being far more frequently the subject of aneurism than those to which it is inapplicable, compression is calculated to supersede the ligature in the great majority of cases.

2. The cure of aneurism by compression upon the artery between the aneurismal sac and the heart, according to the rules laid down here, is accomplished by the gradual deposition of the fibine of the blood in the sac, until both the latter and the artery at the part are completely filled. The process is in fact exactly similar to that by which nature effects a spontaneous cure of aneurism.

3. Such an amount of pressure as would cause inflammation and adhesion between the opposite sides of the artery at the point compressed is never required.

4. The pressure should not be so great as to interrupt the circulation in the artery at the point compressed; an essential agent in the cure being that a current of blood soould pass through the sac.

5. Compression by means of two or more instruments, one of which is alternately relaxed, is much more effectual than by any s ngle instrument, and in many instances the pressure can be maintained by the patient himself.

6. The treatment of aneurism by compression does not involve the slightest risk to the patient, and if persevered in cannot fail of effecting a cure.

7. A cure of aneurism effected by compression, according to the rules laid down here, must necessarily be permanent; and in every case in which a cure has been pccomplished, the patients have remained well subsequently.

8. The femoral artery remains pervious after the cure at the point at which the pressure had been applied, and no morbid change of any kind is to be detected in eitner the artery or vein at the site of the compression.

9. When a cure is effected by compression, the vessel is obliterated only at the seat of the aneurism, and the artery at this part is eventually converted into an impervious ligamentous band.

10. Compression effects the cure of aneurism by more simple and safer means than the ligature, while it is applicable to a number of cases in which the operation is contra-indicated or inadmissible.

11. Compression is not necessarily a more tedious or more painful method of treating aneurism than the ligature, while it is much more certain, more likely to be permanent, and is free from all danger.

12. Compression, according to the rules laid down here, has little analogy with the old method which went by this name; and in fact has no greater resemblance to it than the Hunterian operation had to the operation for aneurism which it superseded.

